# A Retrospective, Observational, EMR-Based Real-World Evidence Study to Assess the Incidence of Pedal Edema in Essential Hypertensive Patients on Amlodipine or Cilnidipine

**DOI:** 10.1155/2022/6868143

**Published:** 2022-02-23

**Authors:** Jamshed Dalal, J. P. Sawhney, P. B. Jayagopal, P. K. Hazra, Mohammed Yunus Khan, Kumar Gaurav, Colette Pinto, Amey Mane, Sachin Rao, Madhur Jain

**Affiliations:** ^1^Cardiac Sciences, Kokilaben Dhirubhai Ambani Hospital, Mumbai, Maharashtra, India; ^2^Department of Cardiology, Sir Ganga Ram Hospital, New Delhi, India; ^3^Lakshmi Hospital, Palakkad, Kerala, India; ^4^AMRI Hospitals, Kolkata, West Bengal, India; ^5^Dr. Reddy's Laboratories Ltd, Hyderabad, Telangana, India; ^6^Cardiology Department, Sri Jayadeva Institute of Cardiovascular Sciences & Research, Mysore, India; ^7^Ujala Cygnus Super Specialty Hospital, Rewari, Haryana, India

## Abstract

**Introduction:**

Calcium channel blockers have pedal edema as one of the confining factors of treatment. A real-world study may help evident reality of the situation in regular Indian clinical practice. The aim of the study is to assess effectiveness and incidence of pedal edema in essential hypertensive patients treated with amlodipine or cilnidipine monotherapy.

**Methods:**

Retrospective EMR data of adult essential hypertensive patients, prescribed amlodipine (*n* = 800) or cilnidipine (*n* = 800) as monotherapy, were analyzed. Incidence of pedal edema from baseline visit was analyzed in terms of dose and duration of treatment. The changes in systolic (SBP) and diastolic blood pressure (DBP) from baseline and proportion of patients achieving target BP goals were assessed.

**Results:**

In amlodipine and cilnidipine groups, mean changes in SBP and DBP from baseline to end of the study period were 28.4 and 15.1 mmHg and 24.3 and 13.5 mmHg, respectively (*p* value <0.05). More than 50% of patients in both groups achieved BP goal at the end of the study (*p* value 0.266). In amlodipine group, total 23.9% reported pedal edema, while in cilnidipine, 27.6% (*p* value 0.0863). At the end of the study, 3.5% and 8.2% of patients remain with pedal edema, respectively, in both groups (*pvalue* <0.005).

**Conclusion:**

Amlodipine demonstrated greater BP reduction at a lower average dose, better efficacy, and tolerability in terms of pedal edema count as a lesser number of patients reported edema at the end of the study and a higher percentage of patients continued the prescribed baseline dosage regimen as compared to cilnidipine. Thus, the study established amlodipine as an effective and well-tolerated antihypertensive for Indians.

## 1. Introduction

Essential hypertension is one of the most common noncommunicable diseases spreading globally with a mortality rate of 31%, as reported in 2016 [1]. The prevalence varies with ethnicity. Fourth District Level Household Survey reported 207 million hypertensive patients in India [2]. Fourth National Family Health Survey with a sample size of 799,228 Indian participants reported hypertension in 13.8% men and 8.8% women [3]. In 2016, the Global Burden of Disease (GBD) study reported 1.63 million deaths, the disease burden of 39 million, and 208 million disability-adjusted life years (DALYs) attributed to hypertension [2–5]. According to the European Society of Cardiology/European Society of Hypertension (ESC/ESH), 2018 Guidelines [6], and Indian Guidelines on Hypertension IV, the authors [7] defined hypertension as the pathophysiological condition with systolic blood pressure (SBP) of ≥140 mmHg and diastolic blood pressure (DBP) of ≥90 mmHg [6–8]. In the treatment of hypertension, the essential step is to control the blood pressure.

Calcium channel blockers (CCB) are proven first-in-line therapies for essential hypertension as monotherapy or in combinations. Amlodipine and cilnidipine are dihydropyridine CCBs prescribed as antihypertensives. The tolerability and efficacy of the two CCBs were established over the years. More significant vasodilation of precapillary vessels as compared to postcapillary vessels is considered as the mechanism attributed to the CCB-induced edema [9]. The prevalence of pedal edema due to CCBs reported to be higher in the warm climate of tropical countries such as India and Brazil [10].

Amlodipine is one of the most preferred drugs of choice for essential hypertension amongst the CCBs. It is owing to its potency (effectiveness), affordability, and long half-life with high bioavailability. The existing literature evidence was mainly generated from random-clinical trials under controlled treatment conditions. Assessing the effectiveness and tolerability of amlodipine in a real-world scenario would establish its usefulness in regular clinical practice in India.

The present retrospective real-world evidence study was designed to assess the incidence of pedal edema and the antihypertensive effectiveness in essential hypertensive patients taking amlodipine or cilnidipine drugs as monotherapy.

## 2. Methodology

### 2.1. Study Design

In this retrospective, longitudinal, real-world, observational study, the electronic medical records (EMR) data of Indian patients diagnosed with essential hypertension from February 2014 to February 2019 were collected. The data were collected from multiple tertiary care centers from the specialties like cardiologists.

### 2.2. Data Sources and Study Sample

The patient EMR were extracted from outpatient hospital records, which were archived in a central medical database used to conduct the analysis.

The adult patients (>18 years) were diagnosed with essential hypertension by their physicians and prescribed amlodipine or cilnidipine as first-line monotherapy and had follow-up data available after initiation (baseline visit) of amlodipine or cilnidipine, included in the study.

Patients diagnosed with secondary hypertension, preexisting edema, and those on other CCBs (except amlodipine or cilnidipine) at visit 1 (baseline) were excluded from the study. Patients with clinically diagnosed conditions such as cor pulmonale, nephrotic syndrome, hypoproteinemia, anemia, severe renal failure, severe heart failure, severe liver failure, and other conditions that can cause edema and pregnant and lactating women were also excluded from the study.

An independent ethics committee (IEC) located in Pune, India, approved the study protocol. This being a retrospective study used the anonymized or anonymous data (existing medical records available as of the date of IEC submission) without any additional prospective components for research purposes. Hence, the process did not necessitate the obligation to obtain informed consent since the study did not involve identifiable individuals. Accordingly, IEC gave permission for the informed consent form waiver before the initiation of the data collection process for this study.

### 2.3. Statistical Analysis

All outcomes presented using descriptive statistics. Continuous data expressed as mean and SD and categorical data as numbers and percentages. The comparison of mean differences of data was analyzed by Mann–Whitney *U* test or *T*-test and categorical variables by the chi-square test. ANOVA was used for testing the significant difference between more than two groups. The *pvalue* <0.05 was considered as statistically significant to determine the difference between the amlodipine and the cilnidipine groups.

#### 2.3.1. Baseline Characteristics

Data consisting of demographic characteristics such as age, gender, personal and family history, and clinical features such as the grade of hypertension as per ESC/ESH 2018 guidelines, BP readings, comorbidities, and concomitant medications were collected at baseline and reported. Blood pressure was measured using a mercury sphygmomanometer. The presence of pedal edema was evaluated by patient complaint, inspection, and general examination.

#### 2.3.2. CCB Effectiveness Analysis

For evaluating effectiveness of the study drugs as antihypertensives, the EMR data having minimum one-month (30 days) gap from the baseline visit (based on available follow-up visits) up to the end of 12 months (study period) were analyzed. The mean changes in SBP and DBP were calculated from the baseline visit to the end of first follow-up visit (1–3 months), second follow-up visit (4–6 months), third follow-up visits (7–9 months), and fourth follow-up visit (10–12 months) (i.e., the end of the study period), or amlodipine or cilnidipine was discontinued, or new therapy was added. While the BP readings of follow-up visits were taken into consideration for assessing the patients reaching target BP goal as per ESC/ESH 2018 guidelines, further details of assessment of mean BP and dose vs mean BP change are given in supporting information.

#### 2.3.3. CCB Tolerability Assessment

For evaluating tolerability, the medical records of patient visits from the baseline to first, second, third, and fourth follow-up visits or whenever patient visited with pedal edema complaint after initiation of amlodipine or cilnidipine treatment, or postindex (amlodipine or cilnidipine discontinuation, the addition of new therapy, or up to end of the study) whichever occurred first, were reviewed. Further details of assessment of pedal edema are given as supporting information.

**Figure 1:**
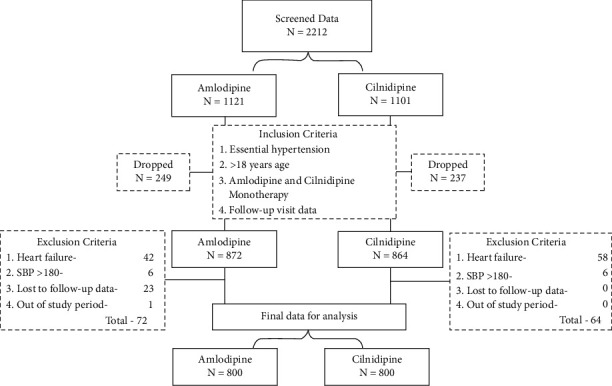
Study sample selection flowchart.

The study sample flowchart is depicted in Figure 1.

## 3. Results

### 3.1. Baseline Parameters

With comparable mean weight, height, and percentages of males and females between the two groups, the highest proportion of patients had grade 1 hypertension (68 and 65%).

In amlodipine group, the duration of hypertension (21 days) was lesser than in the cilnidipine group (44 days). Diabetes, dyslipidemia, and obesity were the three major comorbidities in both groups (Table 1).

### 3.2. Effectiveness of CCB Treatment

The overall average amlodipine dose was 6.6 mg per day and cilnidipine 11.1 mg per day with higher percentage of patients at 5 mg dose (*n* = 479) in amlodipine and at 10 mg (*n* = 384) in the cilnidipine group. In spite of mean dose of cilnidipine being double that of amlodipine, the mean reduction in BP was better with amlodipine in comparison to cilnidipine.

**Figure 2:**
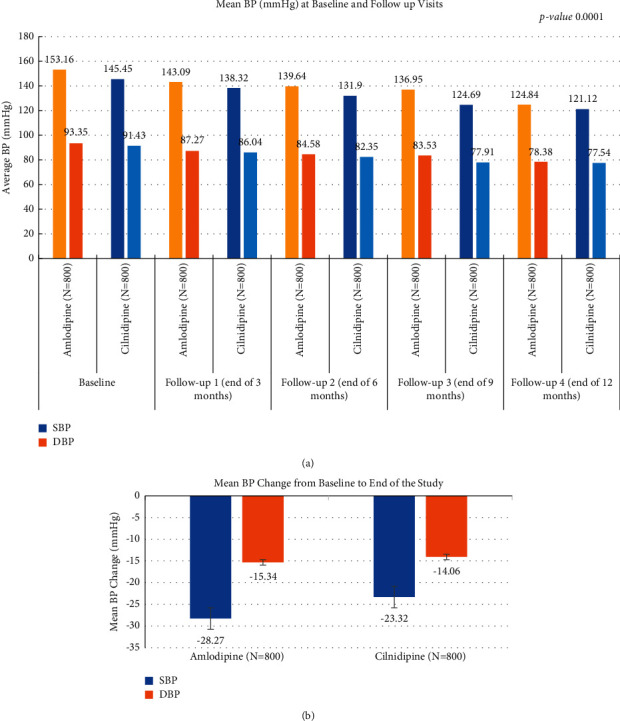
(a) Average BP (mmHg) from baseline to follow-up visits. (b) Mean change in BP from baseline to end of the study.

In the amlodipine group, the SBP and DBP decreased from 153.16 ± 10.17 and 93.35 ± 6.32 mmHg to 124.84 ± 12.24 and 78.38 ± 6.56 mmHg, respectively, during the period of 12 months. The corresponding values for cilnidipine were 145.45 ± 14.71 and 91.43 ± 10.49 mmHg to 121.12 ± 13.19 and 77.54 ± 12.34 mmHg, respectively (Figure 2(a)). The patients with higher BP came out as an observation in the study that in real-world settings, amlodipine is prescribed to the patients with higher BP. This was also ascertained by the study investigators that amlodipine is one of the drugs of choice to control BP in hypertensive patients with higher BP.

Amlodipine decreased the SBP and DBP from baseline to the last follow-up visit to a greater extent (28.4 and 15 mmHg units, respectively) than cilnidipine (24.3 and 13.5 mmHg units) and this difference across the groups was statistically significant (*p* value <0.05) (Figure 2(a)).

#### 3.2.1. Percentage of Patients Achieving BP Goal

At the end of the study, i.e., twelve months from the baseline visit, >50% of the patients could achieve or retain the achieved BP goal in both the groups (*p* value >0.05). During the first to third follow-up visits, 27.0, 35.6, and 36.4% of patients achieved the BP goal in amlodipine group and correspondingly 22.0, 32.0, and 46.9% in cilnidipine group with *p* values 0.048, 0.125, and <0.01, respectively.

#### 3.2.2. Dose vs. Change in BP Reduction

**Figure 3:**
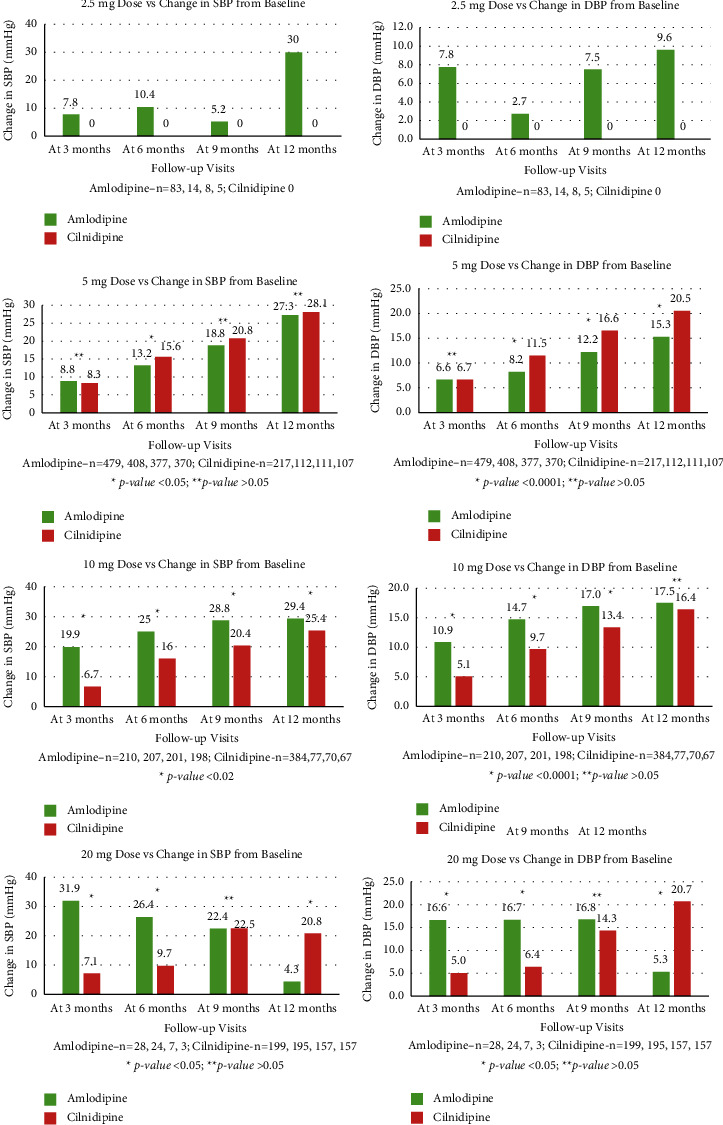
Dose (mg) vs change in BP (mm Hg) from baseline to follow-up visits is given for amlodipine and cilnidipine.

The dose vs change in BP reduction was analyzed from the baseline to follow-up visits for both the groups (Figure 3). In amlodipine, with 2.5 mg dose, the maximum reduction in SBP and DBP was observed at the end of the study period, 30.0 and 9.6 mmHg units, whereas, in cilnidipine, there were no patient on 2.5 mg dosage to draw comparison with amlodipine. The SBP and DBP values at 5 mg dose were 27.3 and 15.3 mmHg units and 28.1 and 20.5 mmHg, for amlodipine and cilnidipine, respectively (*p* value >0.05). At 10 mg dose, at the end of the study, the SBP and DBP were 29.4 and 17.5 mmHg and 25.4 and 16.4 mmHg (*p* value <0.02). At 20 mg, the comparable reduction was observed after the first visit (at the end of 3 months), 31.9 and 16.6 mmHg (*p* value <0.05), whereas, for cilnidipine, at the end of the study (after 9–12 months duration), 20.8 and 20.7 mmHg units (*p* value <0.05).

### 3.3. Tolerability of CCB Treatment

**Figure 4:**
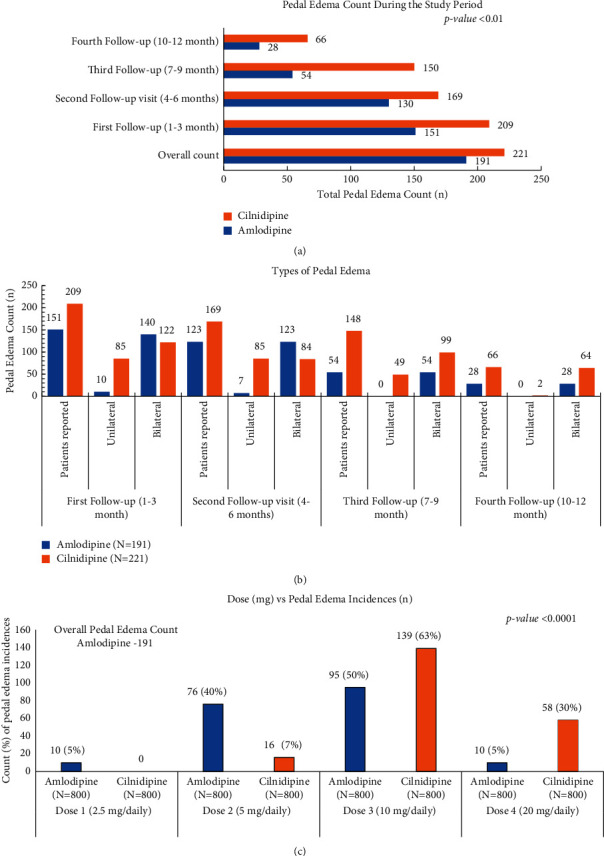
(a) Overall pedal edema count (*n*) reported timelines during the study period. (b) Types of pedal edema count during follow-up visits. (c) Dose (mg) vs pedal edema incidences (*n*).

Overall, at the end of the study, in amlodipine group, 191 (23%) patients reported pedal edema, while in cilnidipine, it was 221 (27.6%) with a *p* value of 0.0863 (Figure 4(a)). Follow-up visit wise, at the end of the first follow-up visit, in amlodipine group, pedal edema was reported in 19% (*n* = 151) patients, which gradually decreased thereafter till the last follow-up visit (3.5%, *n* = 28), while, in cilnidipine group, this percentage decreased from 26% (*n* = 209) at the first follow-up to 8% (*n* = 66) at the last follow-up visit. The intergroup difference was statistically significant (*p* value 0.05) during all four visits (Figure 4(a)).

Overall, the bilateral pedal edema incidences appear to be higher than the unilateral edema. As management of edema condition, CCB was discontinued in 23% and 34% patients, respectively, in both groups (Figure 4(b)).

Overall, the mean duration of onset of pedal edema was 69 days for amlodipine while 51 days for cilnidipine (Figure 4(c)). This was a statistically significant (*p* value = 0.0001) difference across the groups. With a higher proportion of pedal edema reported in 10 mg/daily dose category, the difference in the mean duration of pedal edema between amlodipine (69 ± 50 days) vs cilnidipine (51 ± 32 days) was statistically significant (*p* value <0.05) (Figure 4(c)). At equipotent doses, higher incidences of pedal edema were seen with cilnidipine.

## 4. Discussion

The clinical management of hypertension requires to achieve strict and consistent blood pressure controls through lifelong drug therapy [11, 12]. Each class of antihypertensives carries its advantages and limitations, which compel the treating clinician to carefully choose a better class of drug over the other with a considerable margin of advantage.

A good wealth of evidence has long been proven CCBs as effective antihypertensives, and their use has extended beyond mere control of BP to their impact on cardiovascular safety. Amlodipine has high vascular selectivity, which decreases peripheral resistance while preserving myocardial contractility [13]. Furthermore, amlodipine has an extended elimination half-life. It binds to the target receptors slowly in a sustained manner, leading to a smooth onset of action and controls BP up to 24 hours [14]. Amlodipine not only reduces the risk of cardiovascular events but also decreases the all-cause mortality when compared with non-CCB antihypertensive drugs [15].

Edema is found to be dose-dependent and may exceed 80% with the higher doses of dihydropyridines [16, 17]. Dose titration, drug switchover (i.e., prescribing another antihypertensive drug), and prescribing the combination are the steps for the management of pedal edema. The real-world studies have also reported the same tolerability effects of amlodipine [18–20]. On the other hand, cilnidipine, the newer generation of CCB, inhibits sympathomimetic activity [21]. But different tolerability pattern can be seen between compounds of the same class [22]. Therefore, this real-world study was undertaken to compare the effectiveness and tolerability of amlodipine and cilnidipine.

Khan et al. (2019) in the EMR-based study observed that amlodipine-based regimens reduced SBP (13.3–17.8 mmHg) and DBP (5.5–11.3 mmHg) in patients with essential hypertension [23]. Earlier studies, with patients on amlodipine, reported a mean reduction of 14.6, 15.3, and 17.7 mmHg in SBP and 5.3 and 9.1 mmHg in DBP [24–26]. In the present study, amlodipine, with an average daily dose of 6.6 mg, significantly (*p* value 0.0001) decreased the SBP by 28.3 mmHg and DBP by 15.3 mmHg in 12 months of treatment. The extent of reduction in BP at the last consecutive visits (third to fourth) was also higher in amlodipine group than in cilnidipine group. The gradual decrease in BP over a period of 12 months reflects its long-lasting effectiveness. In spite of the mean dose of cilnidipine being double than that of amlodipine, the mean reduction in BP was better with amlodipine in comparison to cilnidipine.

In an EMR-based study in hypertensive patients on amlodipine, Khan et al. (2019) observed that the ESC/ESH 2018 recommended BP target (≤130/80 mmHg) achieved by 30.1% of patients for SBP and 42.2% for DBP [23]. The proportion of patients achieving the same goal as observed by Bisognano et al. was 16.3% [26], by Ram et al. (2012) in an EMR-based study with 46,706 patients was 21.1% [24], and by Weycker et al. was 45.9% [25]. In the present study, greater percentage of patients achieved this BP goal (≤130/80 mmHg), in amlodipine group (23.4%) at the consecutive visits (third to fourth, i.e., within the last three months of the study). The corresponding percentage of patients in cilnidipine group was 14.5%. Jadhav et al. (2021), in an EMR-based study, comparing amlodipine with other CCBs, on Indian population observed amlodipine as the preferred drug of choice with long-lasting effectiveness and greater effect at lower doses [27]. Amlodipine with longer half-life (*t*_1/2_ = 30–60 hours) than cilnidipine half-life (*t*_1/2_ = 2.5 hours), high bioavailability, and affordability has an added advantage as antihypertensive for Indian population.

Pedal edema is the most common adverse event caused by dihydropyridine CCBs. Previous studies have illustrated the dose-dependent relationship of incidence of edema. Pedal edema is both dose-dependent and molecule specific effect. The incidence of peripheral edema due to CCB varies in the literature due to its dose-dependent nature and among different CCBs [16, 28, 29]. In the present study, the onset of pedal edema was observed in the first three months of treatment in both groups. The duration of onset of pedal edema was in direct correlation with the daily dose, i.e., higher the dose, shorter the duration of onset. At the same dose, a lesser number of patients reported pedal edema with amlodipine than cilnidipine. Greater compatibility was demonstrated by amlodipine as only 5.6% of patients discontinued the treatment amid pedal edema as compared to 9.5% in the cilnidipine group.

## 5. Limitations

This retrospective study, like other retrospective RWE studies, has the drawback that patients themselves were responsible for adhering to and complying with the given prescription. As EMR contains only the prescription data, we cannot exclude the possibility of some patients not adhering to the prescription, leading to one or multiple missed doses, which could have contributed to a low observed therapeutic effect. Different methods were used, instead of a single protocol, to measure the BP at various centers. This may have caused a variation in the measurements. The intersite variability may also be the reason for reported higher pedal edema incidences in the cilnidipine group. The methods used to evaluate the pedal edema have also not been covered in the EMR, so no proper estimation has been made for the same. In the current study, we captured the information of addition of other antihypertensives (other than target drugs) at the end of the study period, i.e., after 12 months. The details included ARB, beta blockers, ACE inhibitors, and alpha blockers, but considering the impact of these changes is beyond the scope of the study since assessing the antihypertensive effect of amlodipine as monotherapy in comparison with cilnidipine is the objective of interest.

## 6. Conclusion

In amlodipine group, higher reduction in SBP and DBP from the baseline to last follow-up visit and to a greater extent in the last consecutive visits (third and fourth) at a lower average dose established better efficacy over cilnidipine. The extent of reduction in BP was observed to be dose-dependent and predictable in amlodipine. Amlodipine showed better tolerability in terms of pedal edema count as a lesser number of patients reported pedal edema at the end of the study period and a higher percentage of patients had continued the prescribed baseline dosage regimen as compared to other groups. Moreover, longer half-life, 24-hour blood pressure control, and greater improvement in CV outcomes are other advantages of amlodipine which make it an antihypertensive drug of choice much suitable for the Indian population.

## Figures and Tables

**Table 1:** Baseline characteristics, comorbidities, and family history.

Basic demographic profile	Amlodipine (*N* = 800)	Cilnidipine (*N* = 800)	*P* value
Age (years) (mean ± SD)	59.42 ± 9.68	54.98 ± 11.37	0.0001
Gender, *n* (%)			
Male	550 (68.75)	535 (66.87)	0.422
Female	250 (31.25)	265 (33.21)	
Weight (Kg) (mean ± SD)	70.02 ± 10.31	71.67 ± 10.70	0.0017
Height (cm) (mean ± SD)	165.00 ± 6.77	164.16 ± 8.12	0.0248
BMI (kg/m^2^) (mean ± SD)	25.65 ± 3.89	26.62 ± 3.65	0.0001
Hypertension complaints reported at baseline			
1. Headache	399 (49.9)	307 (38.4)	-
2. Dizziness	157 (19.6)	53 (6.6)	-
3. Chest pain	149 (18.6)	314 (39.3)	-
4. Breathlessness	108 (13.5)	152 (19.0)	-
5. Fatigue	85 (10.6)	194 (24.3)	-
SBP, mmHg (mean ± SD)	153.05 ± 10.08	145.48 ± 14.71	0.0001
DBP, mmHg (mean ± SD)	93.28 ± 6.27	91.43 ± 10.48	0.0001
Grade of hypertension (as per ESH guidelines), *n* (%)			
Grade 1	545 (68.1)	518 (64.8)	0.161
Grade 2	242 (30.2)	260 (32.5)	
Grade 3	13 (1.6)	22 (2.8)	
Comorbidities and family history			
Details	Amlodipine (*N* = 800) *n* (%)	Cilnidipine (*N* = 800) *n* (%)	*P* value
Without comorbidities	377 (47.13)	221 (27.63)	<0.0001
With comorbidities^@^	423 (52.88)	579 (72.38)	<0.0001
Lifestyle related			
Smokers	160 (20.0)	312 (39.0)	<0.0001
Alcoholics	134 (16.7)	276 (34.5)	<0.0001
Family history of hypertension			
Mother	208 (26.0)	159 (19.9)	0.0035
Father	189 (23.6)	179 (22.4)	0.5524
Other relation	78 (9.7)^#^	16 (2.0)^#^	<0.0001

^*∗*^Amlodipine *n* = 418; cilnidipine *n* = 705. ^@^Diabetes mellitus, dyslipidemia, obesity, heart disease (myocardial infraction, CAD), stroke, kidney disease (eGFR >30 ml/min/1.73 m^2^), anxiety, constipation, hypothyroidism, IBS, joint pain, piles, and prostate disease. ^#^Brother, sister, husband, son, and wife; *T*-test and chi-square were used to calculate the *p* values.

## Data Availability

The data used to support the findings of this study are available for research purpose from the corresponding author upon request.
